# Evaluation of a Self-Driving Shuttle Service to Improve Mobility of Older Adults and People with Disabilities

**DOI:** 10.1093/geroni/igaf122.2012

**Published:** 2025-12-31

**Authors:** Renee St. Louis, Jennifer Zakrajsek, Nicole Zanier, Dillon Funkhouser, James Sayer

**Affiliations:** University of Michigan Transportation Research Institute, Ann Arbor, Michigan, United States; University of Michigan, Ann Arbor, Michigan, United States; University of Michigan, Ann Arbor, Michigan, United States; University of Michigan, Ann Arbor, Michigan, United States; University of Michigan, Ann Arbor, Michigan, United States

## Abstract

This study centers on a one-year pilot program launched in June 2024 in Detroit, Michigan called Accessibili-D. This initiative provides a self-driving shuttle service for individuals aged 62 and older and people with disabilities, addressing traditional transit service gaps and providing an innovative solution to enable continued mobility. Accessibili-D operates six days a week across 128 pre-programmed stops, encompassing medical centers, grocery stores, libraries, and entertainment venues. Participants schedule free rides via a mobile app or by phone. The vehicles operate autonomously on specified routes but are supervised by onboard safety operators. The pilot program enrolled 298 participants in the first three months. Participant evaluations include a baseline survey and three follow-up surveys to assess travel behavior, shuttle utilization, trust in automated vehicle (AV) technologies, and opinions on how to improve the service. Results to date revealed that those who have ridden the shuttle agreed more strongly than non-riders that AVs are dependable (χ2=12.61, p=.01), reliable (χ2=13.73, p=.01), that they can trust AVs (χ2=12.18, p=.02), and that AVs will be useful in meeting their transportation needs (χ2=10.27, p=.04). Key findings from the qualitative analysis of open-text responses revealed the desire for an expanded service area and the importance of the safety operator to the participants’ riding comfort. Details of the shuttle service and outcomes of the baseline and follow up surveys to address participant experience and changes over time will be discussed. Insights from this pilot will inform scalable approaches to AV deployment, promoting safer and more equitable transportation systems.

